# Editorial: Coffee and coffee by-products: innovative approaches fostering nutritional, sensory and chemical quality

**DOI:** 10.3389/fnut.2024.1542109

**Published:** 2025-01-07

**Authors:** Nurhan Uslu

**Affiliations:** Food Engineering Department, Faculty of Agriculture, Selçuk University, Konya, Türkiye

**Keywords:** bioactive properties, coffee, sensory attributes, physicochemical properties, coffee by-products

Coffee, in addition to being one of the most widely consumed beverages, is considered a functional food because it contains antioxidant and health-beneficial compounds (1, 2). Several factors such as species, location, cultivation method, climate conditions affect the chemical composition of coffee beans (3). In addition, processing parameters, such as roasting degree, extraction time, water composition and temperature, pressure, particle size, and the coffee/water ratio, are of great importance in the aroma, quality and consumer acceptance of coffee (4). Moreover, the coffee brewing methods also attract considerable attention (5). These are very important for sensory properties, nutritional characteristics, antioxidant activities, and volatile and non-volatile compounds of coffee. The coffee extraction process basically consists of three stages: (1) water absorption of ground coffee, (2) transfer of soluble components from coffee to water, (3) separation of extract from grounds (6). Coffee brewing methods vary depending on cultural habits, social environment and personal preferences, but the ultimate goal is to produce a high-quality beverage (4). Coffee quality also considers the control of contaminants in both green and roasted coffee. Together with these, the concentration of caffeine and its dependence on bean origin, variety, altitude, and post-harvesting processes, must be considered in order to get low-caffeine coffee that avoids chemical treatment of green beans that may lead to a reduction in sensory and nutritional qualities. Therefore, investigating post-harvest conditions and determining optimum processes are necessary for the production of healthy and high-quality coffee. In addition, there is a need to valorize coffee by-products along the entire supply chain that are often rich in high-value nutritional compounds. Green and mild processing technologies should be considered to valorize by-products in order to preserve their nutritional values, functionalities, and to ensure their usability in food applications.

This Research Topic is aimed to investigate new methods, processes and technologies both to detect the quality of coffee in the process from the green bean to the beverage and to valorize the by-products.

Within the scope of this Research Topic, there are four articles investigating different aspects of coffee. In the first study, the relationship between caffeinated and decaffeinated coffee consumption and mortality in elderly people with different cognitive performances was examined and it was stated that the relationship between coffee consumption and mortality was affected by cognitive abilities (Lin et al.). The effects of systems consisting of different management practices (conventional and organic) and shade types on the physical and chemical properties of coffee beans were investigated and it was reported that considering sustainability, IO management practice associated with Erythrina shade tree system would be a beneficial combination for local farmers to grow coffee trees (Xu et al.). In another study, the macronutrient and carotenoid contents of red and yellow *Coffea arabica* var. Caturra pulp, a coffee by-product from Colombia, were investigated and alternative solutions for the evaluation of this by-product were presented (Rojas-Orduña et al.). In the last study, the effects of cultivation area and roasting process on the biochemical composition of Ethiopian arabica coffee beans were investigated and it was determined that both location and roasting process had significant effects on the bioactive components of coffee (Mengesha et al.).

In conclusion, the above mentioned studies provide new information on the physicochemical properties, bioactive components and health effects of coffee and its by-products. There is a need for up-to-date data on the cultivation and processing of coffee, whose consumption is increasing day by day, and the evaluation of its by-products in the food sector. In addition, it is recommended that new technologies be used in the stages of preparing coffee beans for beverages, and thus more detailed studies be carried out to produce higher quality coffee while preserving the health-beneficial compounds.
